# Wnt5A Signaling Regulates Gut Bacterial Survival and T Cell Homeostasis

**DOI:** 10.1128/msphere.00507-22

**Published:** 2022-12-06

**Authors:** Soham Sengupta, Suborno Jati, Shreyasi Maity, Malini Sen

**Affiliations:** a Division of Cancer Biology & Inflammatory Disorder, CSIR-Indian Institute of Chemical Biology, Kolkata, West Bengal, India; b Department of Chemistry and Biochemistry, University of California-San Diego, La Jolla, California, USA; University of Michigan-Ann Arbor

**Keywords:** actin, commensal bacteria, T cell, Wnt5A

## Abstract

In light of the demonstrated antagonism of Wnt5A signaling toward the growth of several bacterial pathogens, it was important to study the influence of Wnt5A on gut-resident bacteria and its outcome. Here, we demonstrate that in contrast to inhibiting the survival of the established gut pathogen Salmonella enterica, Wnt5A clearly promotes the survival of the common gut commensals Enterococcus faecalis and Lactobacillus rhamnosus within macrophages through a self-perpetuating Wnt5A-actin axis. A Wnt5A-actin axis furthermore regulates the subsistence of the natural bacterial population of the Peyer’s patches, as is evident from the diminution in the countable bacterial CFU therein through the application of Wnt5A signaling and actin assembly inhibitors. Wnt5A dependency of the gut-resident bacterial population is also manifested in the notable difference between the bacterial diversities associated with the feces and Peyer’s patches of Wnt5A heterozygous mice, which lack a functional copy of the Wnt5A gene, and their wild-type counterparts. Alterations in the gut commensal bacterial population resulting from either the lack of a copy of the Wnt5A gene or inhibitor-mediated attenuation of Wnt5A signaling are linked with significant differences in cell surface major histocompatibility complex (MHC) II levels and regulatory versus activated CD4 T cells associated with the Peyer’s patches. Taken together, our findings reveal the significance of steady state Wnt5A signaling in shaping the gut commensal bacterial population and the T cell repertoire linked to it, thus unveiling a crucial control device for the maintenance of gut bacterial diversity and T cell homeostasis.

**IMPORTANCE** Gut commensal bacterial diversity and T cell homeostasis are crucial entities of the host innate immune network, yet the molecular details of host-directed signaling pathways that sustain the steady state of gut bacterial colonization and T cell activation remain unclear. Here, we describe the protective role of a Wnt5A-actin axis in the survival of several gut bacterial commensals and its necessity in shaping gut bacterial colonization and the associated T cell repertoire. This study opens up new avenues of investigation into the role of the Wnt5A-actin axis in protection of the gut from dysbiosis-related inflammatory disorders.

## INTRODUCTION

Wnt5A belongs to a family of glycoprotein ligands that initiate signaling upon binding to the Frizzled and ROR/Ryk families of transmembrane cell surface receptors. While Frizzleds resemble the heterotrimeric G protein-coupled receptors, ROR1, ROR2, and Ryk resemble tyrosine kinases (1–3). Wnt-Frizzled/ROR/Ryk signaling has long been known to be crucial for tissue patterning and organism development (4–7). Through the course of research, it has now become clear that Wnt signaling also features as a prominent player in infection, inflammation, and immunity in different modes that are to a large extent context dependent (8–11).

Wnt signaling operates as two major pathways—β-catenin dependent (canonical) and β-catenin independent (noncanonical). Wnt-directed canonical signaling pathways almost invariably involve the transcriptional activation of β-catenin-Lef/Tcf responsive genes, whereas Wnt directed noncanonical signaling pathways often operate independently of β-catenin. Although Wnt5A signaling represents the noncanonical mode of Wnt signaling as per classification, due to the existing homology among the different Wnt cognate receptors and the presence of common Wnt signaling intermediates, there is overlap of Wnt5A signaling with the canonical Wnt signaling pathway (12–14).

In compliance with the involvement of Wnt5A signaling in organelle trafficking and cell polarity (7, 15, 16), we demonstrated along with others the role of Wnt5A in actin remodeling and phagocytosis of foreign matter, including microbes (8, 17, 18). We also demonstrated that Wnt5A-regulated actin organization directs the killing of microbial pathogens through the generation of autophagosome-like moieties within macrophages (19). These findings led us to examine the interrelation between Wnt5A signaling and the commensal bacteria that form part and parcel of human physiology.

Among other anatomical niches within the mammalian host, the Peyer’s patches of the gut are specially noted for harboring phagocytes and a multitude of commensal bacteria that are crucial for the maintenance of gut immune homeostasis (20–23). Quite naturally, loss or change in the bacterial population leading to alterations in bacterial diversity is linked with the development of several autoimmune disorders (24). Although an interrelation between Wnt expression and intestinal commensal bacterial colonization has been suggested (25), much remains unknown about whether or how Wnt signaling in the steady state controls the intestinal bacterial population, thereby influencing gut immunity.

In the current study, using mouse macrophages, isolated mouse Peyer’s patches, and a mouse model, we examined if Wnt5A signaling is involved in the maintenance of gut microbial diversity and the T cell activation profile. Our experimental results reveal that Wnt5A signaling aids in the preservation of gut commensal bacterial colonization and CD4 T cell homeostasis.

(This article was submitted to an online preprint archive [26].)

## RESULTS

### Wnt5A signaling promotes uptake and survival of common gut commensal bacteria by phagocytes.

**FIG 1:**
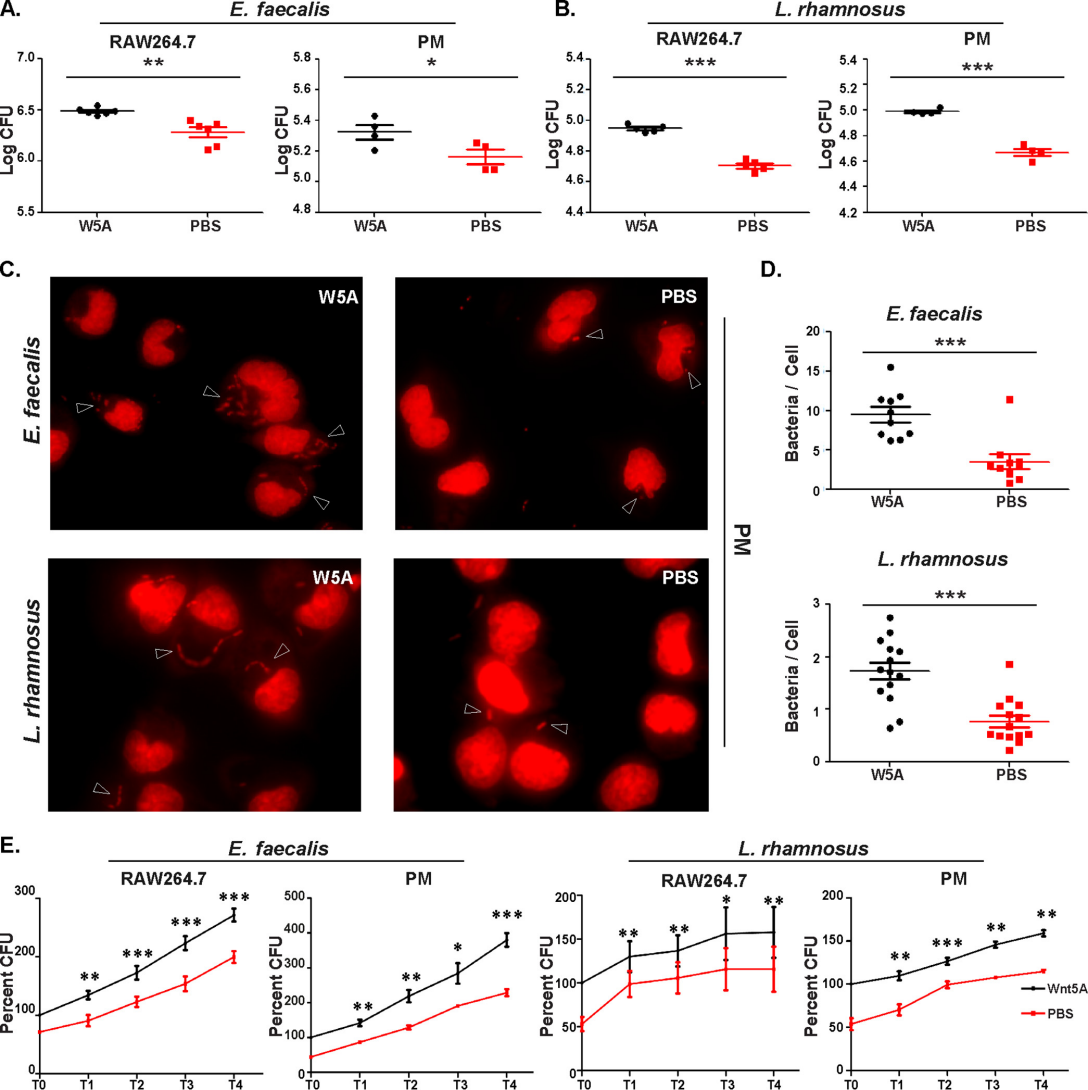
Wnt5A (W5A) aided internalization and survival of bacterial commensals. (A) Pretreatment of RAW264.7 (*n* = 6) and peritoneal macrophages (PM) (*n* = 4) with rWnt5A for 6 h led to higher internalization of E. faecalis after 2 h of infection at an MOI of 10, as evident from the CFU. (B) A similar increase in internalization of L. rhamnosus (enumerated by CFU) following infection (MOI, 20) of rWnt5A-pretreated RAW264.7 (*n* = 5) and peritoneal macrophages (*n* = 4). CFU enumerated as log (CFU × dilution factor). (C) E. faecalis and L. rhamnosus internalization in peritoneal macrophages (rWnt5A versus PBS pretreated) as observed by fluorescence microscopy of propidium iodide-stained cells. (D) Enumeration of the number of bacteria/cell as observed in different microscopy fields (*n* = 10 to 14 fields). (E) Wnt5A-mediated survival/proliferation of internalized E. faecalis and L. rhamnosus (*n* = 3 to 4) as enumerated by CFU 1 to 4 h (*T*1 to *T*4) postinfection (*T*0). Data are represented as the mean ± SEM. *, *P* ≤ 0.05; **, *P* ≤ 0.005; ***, *P* ≤ 0.0005; n, number of experiments unless stated otherwise.

**FIG 2:**
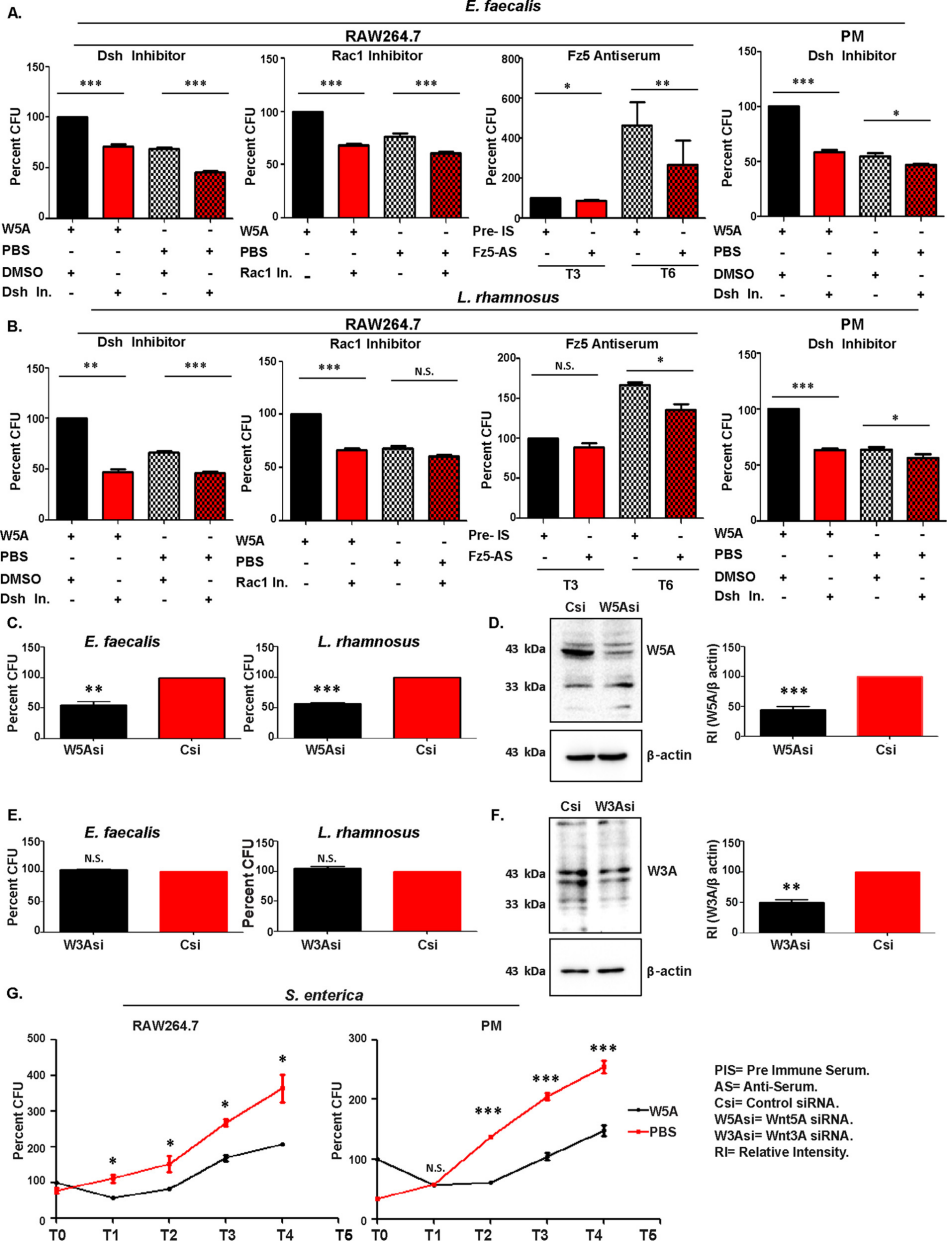
Inhibition of Wnt5A signaling blocks survival of commensal bacteria. (A and B) Application of inhibitors of Wnt5A signaling intermediates Dsh and Rac-1 for 3 h, as well as blocking of Fz5 receptor by application of antiserum for 3 and 6 h postinfection by E. faecalis (MOI, 10 for 1 h) (A) and L. rhamnosus (MOI, 20) (B) led to a CFU reduction in infected RAW264.7 or PM cells (*n* = 3 to 6). (C) Knockdown of Wnt5A by siRNA postinternalization of E. faecalis and L. rhamnosus led to a decrease in bacterial survival as enumerated by CFU (*n* = 4). (D) Immunoblot and densitometry showing a decrease of Wnt5A in Wnt5A siRNA-transfected cells compared to the control. (E) Downregulation of Wnt3A by siRNA did not have a significant effect on bacterial survival (*n* = 4). (F) Immunoblot and densitometry confirming siRNA-mediated Wnt3A depletion. (G) Upregulation of Wnt5A signaling opposed the survival of internalized S. enterica (*n* = 3 for both RAW264.7 and peritoneal macrophages). Data are represented as the mean ± SEM. A *P* value of ≤0.05 was considered statistically significant. *, *P* ≤ 0.05; **, *P* ≤ 0.005; ***, *P* ≤ 0.0005; N.S., nonsignificant.

In order to evaluate the influence of Wnt5A signaling on the cellular uptake and survival of gut commensal bacteria, we initially focused on two common gut commensals, Enterococcus faecalis and Lactobacillus rhamnosus, using the macrophage line RAW264.7 and peritoneal macrophages, which comprise different types of cells, as representative phagocytes (27–30). The Enterococcus faecalis strain was identified through screening of the mouse cecum-resident bacteria by Wnt5A-mediated augmented internalization into macrophages and later validated by 16S sequencing (see Fig. S1A and B in the supplemental material). Wnt5A-mediated augmented uptake of E. faecalis by RAW264.7 and mouse peritoneal macrophages was subsequently confirmed by enumerating the CFU from independent uptake experiments using recombinant Wnt5A (rWnt5A) (Fig. 1A). Uptake was enumerated as log (CFU × dilution factor) and denoted as log CFU. Lactobacillus rhamnosus purchased commercially and further validated by 16S sequencing (Fig. S1C) was also internalized in higher numbers by the same cells in response to added Wnt5A compared to the corresponding vehicle (phosphate-buffered saline [PBS]) control (Fig. 1B). Augmented uptake of both E. faecalis and L. rhamnosus in the Wnt5A-activated peritoneal macrophages was also demonstrated separately by microscopy (Fig. 1C and D). Continued incubation of the Wnt5A-activated infected cells for several hours following bacterial uptake (zero time point: T0) indicated that increased uptake correlates with increased survival/proliferation of the internalized bacteria (Fig. 1E). That Wnt5A signaling facilitates the survival of these commensals was further corroborated by the observed attenuation in bacterial CFU through application of inhibitors of the Wnt5A signaling intermediates Disheveled (Dsh) (31, 32) and Rac1 (33, 34) and anti-Frizzled5 antiserum (35) to the Wnt5A-activated bacteria harboring RAW264.7 cells, which were left to incubate for 3 (T3) or 6 (T6) h following infection. Similar results were obtained with bacteria harboring peritoneal macrophages through the application of Dsh inhibitor (Fig. 2A and B). An inhibitor-mediated decrease in bacterial counts in the absence of added Wnt5A (PBS samples) clearly suggested that Wnt5A signaling at the steady state is involved in bacterial survival. A requirement of steady state Wnt5A signaling for bacterial survival was validated by the loss of bacterial CFU in the infected RAW264.7 cells, where the Wnt5A level was reduced by Wnt5A small interfering RNA (siRNA) transfection (Fig. 2C). Reduction of Wnt5A was evident from immunoblotting and subsequent densitometry (Fig. 2D). Wnt3A reduction did not have any significant effect on the survival of E. faecalis and L. rhamnosus as demonstrated by similar experiments, indicating that there is considerable specificity in the interrelation between these gut commensals and Wnt5A signaling (Fig. 2E and F). The prosurvival effect of Wnt5A on E. faecalis and L. rhamnosus was in stark contrast to its antagonism toward the growth of the gut pathogen Salmonella enterica in both RAW264.7 and peritoneal macrophages (Fig. 2G). The nonpathogenic nature of E. faecalis and L. rhamnosus, both of which formed biofilms, was supported by their vancomycin sensitivity. S. enterica, the gut pathogen, demonstrated vancomycin resistance (Fig. S1D to H).

10.1128/msphere.00507-22.1 FIG S1 Isolation, identification, and characterization of Wnt5A-responsive bacterial commensals. (A) Bacteria isolated from the cecum of BALB/c mice were tested for responsiveness toward Wnt5A signaling by estimating their internalization by RAW264.7 cells pretreated with rWnt5A or PBS (vehicle control). Isolates were named B1 to B5 for convenience of identification. Isolates B3 and B5 showed the highest responsiveness toward Wnt5A signaling (*n* = 3 to 8). The CFU count is represented as CFU (× dilution factor). (B) Isolate B3 was identified as E. faecalis by 16S sequencing. (C) The identity of commercially acquired L. rhamnosus was also verified by 16S sequencing. (D and E) E. faecalis (D) and L. rhamnosus (E) displayed biofilm forming potential, a common feature reported in gut commensals. Biofilm formation was observed by crystal violet (CV) staining (*n* = 3). (F and G) E. faecalis (F) and L. rhamnosus (G) lacked pathogenicity as confirmed by lack of vancomycin resistance. Newly inoculated bacterial culture failed to grow, and grown culture died out when the CDC-recommended 6 μg/mL vancomycin was added in growth medium and observed with respect to the control (without vancomycin) (*n* = 3). (E) S. enterica, a well-established gut pathogen displayed vancomycin resistance (*n* = 3). Data are represented as the mean ± SEM. A *P* value of ≤0.05 was considered statistically significant. *, *P* ≤ 0.05; **, *P* ≤ 0.005; ***, *P* ≤ 0.0005; N.S., nonsignificant; n, number of experiments. Download FIG S1, TIF file, 0.2 MB. Copyright © 2022 Sengupta et al. 2022 Sengupta et al. https://creativecommons.org/licenses/by/4.0/ This content is distributed under the terms of the Creative Commons Attribution 4.0 International license.

### A Wnt5A-actin axis supports the survival of commensal bacteria.

**FIG 3:**
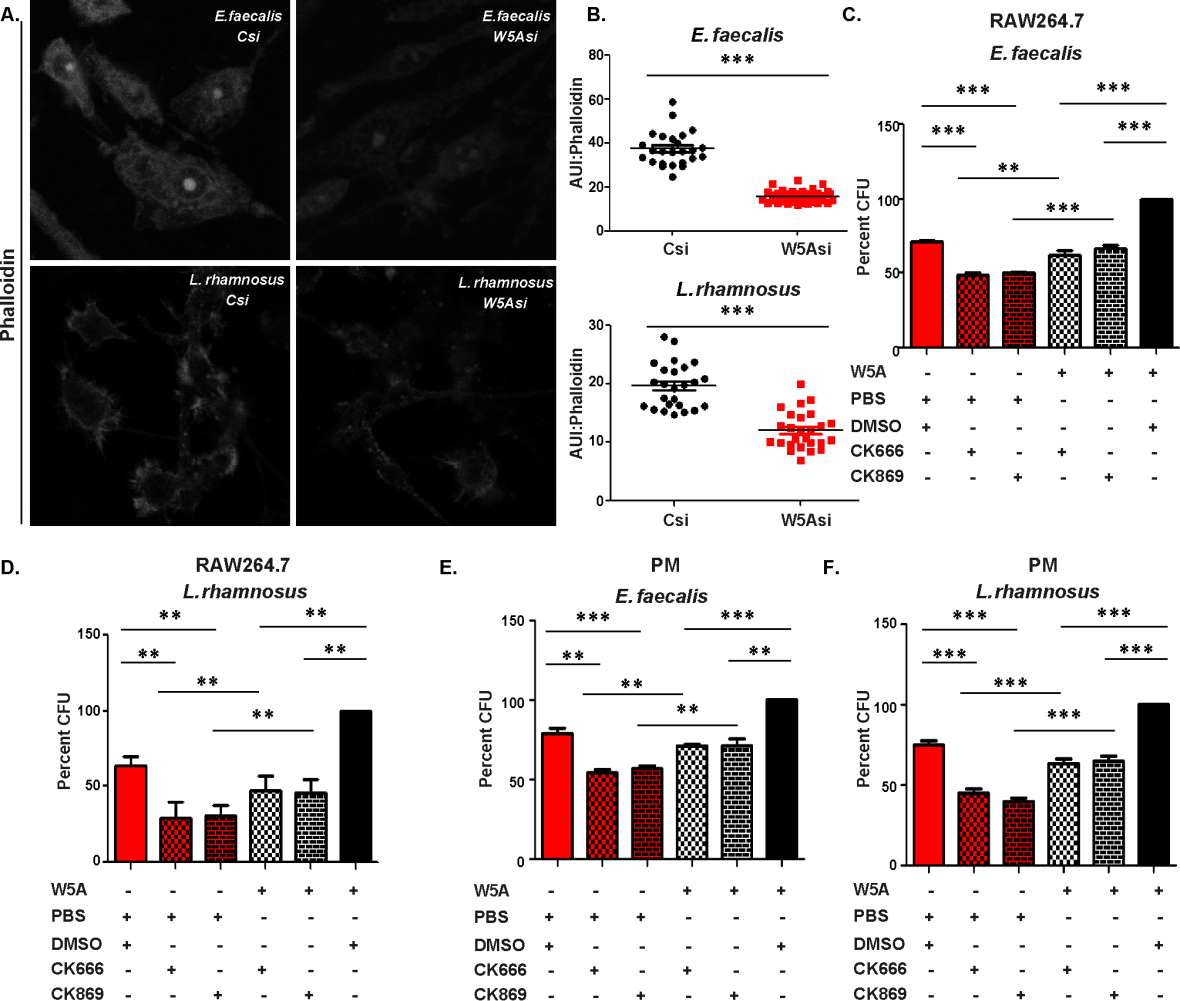
The Wnt5A-actin axis supports survival of commensal bacteria. (A) Wnt5A downregulation by siRNA led to decreased actin accumulation in RAW264.7 cells infected with E. faecalis and L. rhamnosus as noted from confocal microscopic images of phalloidin-stained cells. Images are represented in monochrome. (B) Enumeration of arbitrary unit of intensity (AUI) of phalloidin staining as a measure of actin assembly (*n* = 25 cells). (C to F) Application of actin polymerization inhibitors CK666 and CK869 inhibited survival of internalized E. faecalis and L. rhamnosus in RAW264.7 cells (C and D) and peritoneal macrophage cells (E and F), which was considerably recovered by the addition of rWnt5A. rWnt5A without actin assembly inhibitors promoted maximum survival (*n* = 4). Data are represented as the mean ± SEM. A *P* value of ≤0.05 was considered statistically significant. *, *P* ≤ 0.05; **, *P* ≤ 0.005; ***, *P* ≤ 0.0005.

**FIG 4:**
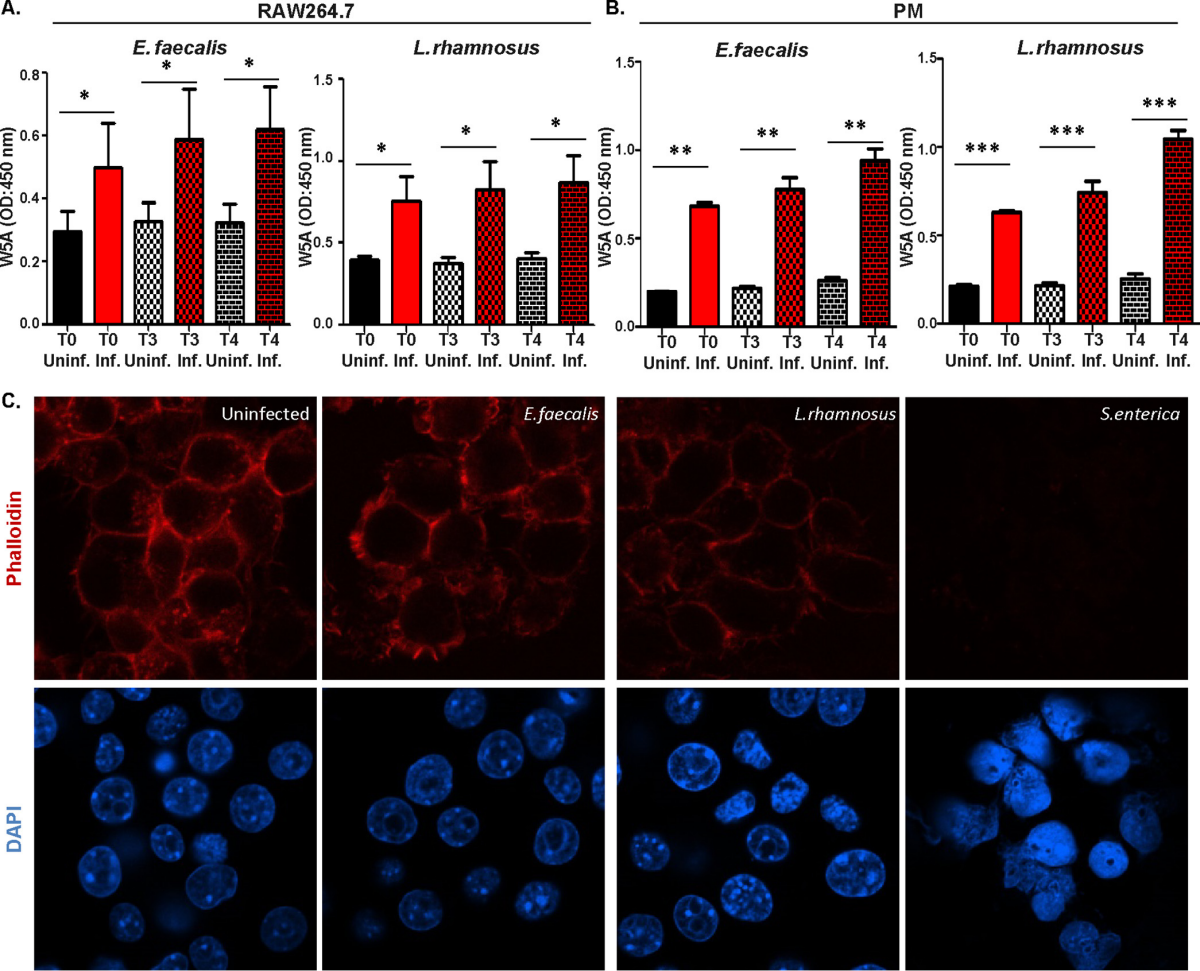
Wnt5A secretion and actin assembly in commensal bacteria-harboring phagocytes. (A) Infection of RAW264.7 cells by E. faecalis and L. rhamnosus increased secretion of Wnt5A 3 and 4 h (*T*3 and *T*4) postinfection (*T*0) as observed by indirect ELISA (*n* = 5). (B) A similar increase in Wnt5A secretion was observed from infected peritoneal macrophage cells (*n* = 3). (C) Infection with E. faecalis and L. rhamnosus maintained actin assembly as seen from phalloidin staining of RAW264.7 cells. The pathogen S. enterica severely hampered actin organization. Data are represented as the mean ± SEM. A *P* value of ≤0.05 was considered statistically significant. *, *P* ≤ 0.05; **, *P* ≤ 0.005; ***, *P* ≤ 0.0005.

In light of the demonstrated association of actin dynamics with Wnt5A signaling (9, 17, 36), we investigated if Wnt5A-assisted survival of the commensal bacteria is linked with actin assembly. Indeed, Wnt5A depletion-induced reduction in E. faecalis and L. rhamnosus survival in macrophages was clearly associated with diminution in actin assembly as demonstrated by confocal microscopy and subsequent ImageJ (intensity) analysis of phalloidin-stained cells (Fig. 3A and B). The necessity of actin assembly for both E. faecalis and L. rhamnosus survival, and the involvement of Wnt5A therein, was separately demonstrated by actin assembly inhibitor-mediated loss of bacterial CFU that was considerably recovered by the addition of rWnt5A to the inhibitor-treated E. faecalis and L. rhamnosus harboring RAW 264.7 cell cultures during a 3-h incubation period following infection. The significant gain in bacterial CFU by the addition of rWnt5A in the absence of actin assembly inhibitors further confirmed the prosurvival effect of the Wnt5A-actin axis (Fig. 3C and D). A similar need for the Wnt5A-actin axis for E. faecalis and L. rhamnosus survival was also demonstrated in peritoneal cells (Fig. 3E and F). Interestingly, E. faecalis or L. rhamnosus internalization (*T*0: 0 h) and continued incubation of the infected macrophages (*T*3, *T*4: 3 h, 4 h) resulted in increased secretion of Wnt5A that correlated with perfect maintenance of actin assembly. The intactness of actin assembly in the E. faecalis- or L. rhamnosus-infected macrophages was clearly distinct from the almost dilapidated state of actin in the S. enterica-infected macrophages (Fig. 4). These observations point toward a coordinated self-sustaining circuit of Wnt5A signaling (autocrine/paracrine), actin assembly, and bacterial survival in the E. faecalis- or L. rhamnosus-infected, but not S. enterica-infected cells (Fig. S2). Such a concept is in agreement with the observed blockade in E. faecalis or L. rhamnosus survival by the administration of anti-Frizzled5 antiserum and other Wnt5A signaling inhibitors to the bacteria-harboring phagocytes (Fig. 2).

10.1128/msphere.00507-22.2 FIG S2 Model depicting Wnt5A-actin axis-mediated survival of bacterial commensals through a self-perpetuating Wnt5A-actin-bacteria circuit. (A) Wnt5A supports both internalization and survival of bacterial commensals, which are in harmony with the Wnt5A-actin axis. Commensal internalization enhances Wnt5A secretion, leading to a self-sustaining Wnt5A-actin assembly-commensal survival loop. (B) Wnt5A upregulates internalization of the pathogen S. enterica, but the pathogen disrupts the host actin machinery, causing Wnt5A signaling to antagonize its survival. Animations were generated using Biorender (Agreement no.- KJ24JTGSU5 and BV24JTISR2). Download FIG S2, TIF file, 0.2 MB. Copyright © 2022 Sengupta et al. 2022 Sengupta et al. https://creativecommons.org/licenses/by/4.0/ This content is distributed under the terms of the Creative Commons Attribution 4.0 International license.

### Wnt5A signaling regulates the growth/colonization of commensal bacteria in the mouse gut.

**FIG 5:**
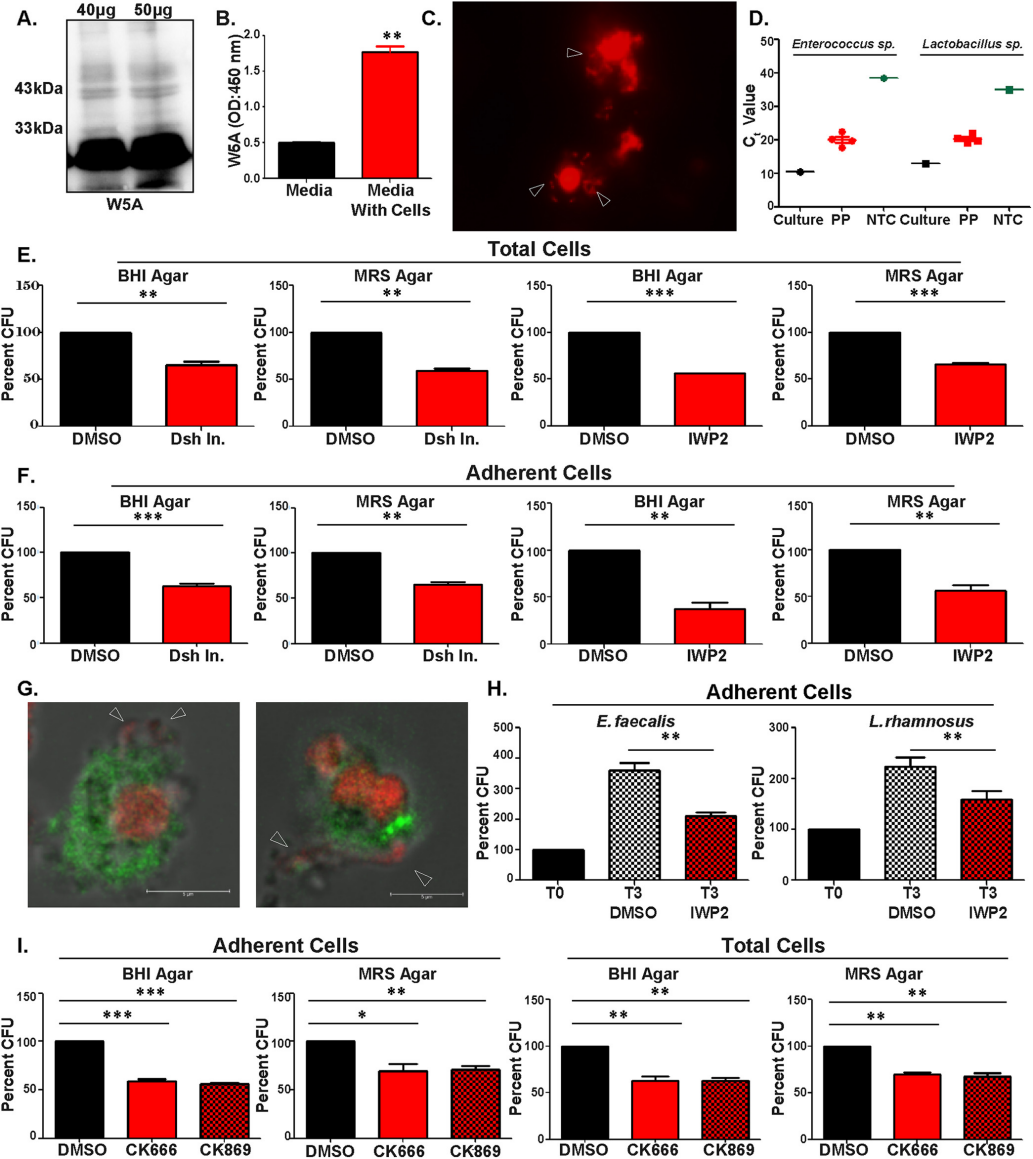
Wnt5A signaling influences commensal bacterial survival in the Peyer’s patches. (A and B) Wnt5A expression in total cells of Peyer’s patches of BALB/c mice by immunoblot (A) and ELISA of cell supernatant (B) (*n* = 2 to 3). (C) Intracellular bacteria in the adherent cells of Peyer’s patches as observed by fluorescence microscopy after PI staining. (D) Estimation of *Enterococcus* spp. and *Lactobacillus* spp. in Peyer’s patches by qPCR (*n* = 4 mice); NTC indicates no-template control, and “culture” (E. faecalis or L. rhamnosus) is used as the positive control. (E and F) Application of Dsh and Wnt5A production inhibitor IWP2 for 6 h led to diminution of countable bacterial CFU from total cells (E) and adherent cells (F) of Peyer’s patches (*n* = 3 to 4). (G) Bacteria (PI, red), denoted by arrowhead, associated with CD11b+ (green) phagocytes of Peyer’s patches as observed by confocal microscopy. (H) IWP2 blocked survival of E. faecalis and L. rhamnosus in adherent cells of Peyer’s patches as observed from CFU 3 h (*T*3) postinternalization (*T*0) (*n* = 4). (I) Administration of Arp 2/3 complex inhibitor I (CK666) and Arp 2/3 complex inhibitor II (CK869) for 6 h to cells of Peyer’s patches led to a decrease in endogenous CFU (*n* = 3). Data are represented as the mean ± SEM, and a *P* value of ≤0.05 was considered statistically significant. *, *P* ≤ 0.05; **, *P* ≤ 0.005; ***, *P* ≤ 0.0005.

In view of the fact that the Peyer’s patches of the small intestine constitute a major hub for gut-resident commensal bacteria and phagocytes (21, 22, 37–39), we investigated the influence of Wnt5A signaling on the growth/colonization of commensal bacteria therein. Initially, we demonstrated that the Peyer’s patches of the mouse gut express and secrete Wnt5A by both immunoblotting and enzyme-linked immunosorbent assay (ELISA) (Fig. 5A and B) and linked Wnt5A expression to different kinds of phagocytes by fluorescence-activated cell sorting (FACS). The phagocyte profile was found to be quite similar to that of Wnt5A expressing RAW264.7 and peritoneal macrophages (Fig. S3). Subsequently, we confirmed the existence of intracellular bacteria (depicted by arrowheads) by microscopy of cultured adherent phagocytes of the Peyer’s patches through propidium iodide (PI) staining (Fig. 5C). The presence of *Enterococcus* spp. and *Lactobacillus* spp. in the Peyer’s patches was ascertained by both quantitative PCR (qPCR) of RNA isolated from harvested tissue and PCR of genomic DNA isolated from cultured adherent phagocytes (Fig. 5D and Fig. S4). Subsequently, we demonstrated that modulation of Wnt5A signaling affects bacterial survival in the Peyer’s patches. Figure 5E depicts a significant reduction in the brain heart infusion (BHI)/de man rogosa and sharpe (MRS) agar-grown bacterial colonies harvested from the total cells of the Peyer’s patches following incubation of the cells for 6 h separately with IWP-2 and Dsh inhibitor. Dimethyl sulfoxide (DMSO) was used as the vehicle control in these assays. The potency of the Dsh inhibitor was verified by the inhibition of Wnt5A-facilitated bacterial internalization in macrophages, and the potency of IWP-2 was confirmed by blockade of Wnt5A secretion. Application of the chemical inhibitors did not induce any noticeable change in cell counts or morphology (Fig. S5). An inhibitor-mediated alteration in the number of countable bacterial colonies was also reflected in the adherent cells of the Peyer’s patches (Fig. 5F), which were noted, considering the adherent tendencies of the different phagocytes (CD11c+/CD11b+/Ly6c+) as observed by FACS of isolated Peyer’s patches (40) (Fig. S3). The presence of PI-stained intracellular bacteria (red, depicted by arrowhead) demonstrated in Fig. 5C was additionally verified in the context of CD11b+ (green) phagocytes by confocal microscopy (Fig. 5G). The same inhibitor IWP-2 also blocked the survival of E. faecalis and L. rhamnosus after their internalization (*T*0) in the adherent phagocytes of the Peyer’s patches during a 3-h (*T*3) incubation period (Fig. 5H). These adherent cells were pretreated with antibiotic for removal of endogenous bacteria. In accordance with the inherent association of actin assembly with Wnt5A signaling, we furthermore found that application of actin assembly inhibitors led to a significant reduction in the number of BHI/MRS agar-grown bacterial colonies harvested from the isolated cells of the Peyer’s patches (Fig. 5I). Actin assembly inhibitors did not cause any noticeable reduction in the number or morphology of the cells isolated from the Peyer’s patches (Fig. S5). Adherent cells were studied to evaluate phagocyte-T cell association in the context of bacterial commensalism, as explained later in the text. Total cells were considered in addition to the adherent cells so as to not miss out on bacteria harboring cells that are weakly adherent.

10.1128/msphere.00507-22.3 FIG S3 Peyer’s patches comprise Wnt5A-expressing phagocyte subsets similar to peritoneal cells and RAW264.7 cells. (A) FACS plots demonstrating Wnt5A (W5A)-expressing phagocytes in Peyer’s patches mostly belong to CD11c+, CD11b+, and Ly6C+ subsets. The possibility of overlapping expression of markers can be noted from the plots. (B and C) Similar phagocyte populations with similar marker expression were also noted from cells in the peritoneal exudates (B) as well as the RAW264.7 cell line (C). Wnt5A was gated using the secondary control; all other gates were placed on the basis of unstained cells. Download FIG S3, TIF file, 0.2 MB. Copyright © 2022 Sengupta et al. 2022 Sengupta et al. https://creativecommons.org/licenses/by/4.0/ This content is distributed under the terms of the Creative Commons Attribution 4.0 International license.

10.1128/msphere.00507-22.4 FIG S4 Bacterial commensals are present within the cells of the Peyer’s patches. (A) E. faecalis identified in cells of Peyer’s patches upon PCR amplification of isolated genomic DNA with E. faecalis-specific primers. (B) Identification of L. rhamnosus in the cells of Peyer’s patches in a similar manner. Download FIG S4, TIF file, 0.04 MB. Copyright © 2022 Sengupta et al. 2022 Sengupta et al. https://creativecommons.org/licenses/by/4.0/ This content is distributed under the terms of the Creative Commons Attribution 4.0 International license.

10.1128/msphere.00507-22.5 FIG S5 Verification of Wnt5A signaling and actin polymerization inhibitors. (A and B) Application of 0.05 μM IWP2 inhibited the secretion of Wnt5A (W5A) but not Wnt3A (W3A) from total cells of Peyer’s patches as observed by indirect ELISA (*n* = 5, *n* = 4). (C) Application of 15 μM Dsh inhibitor in combination with rWnt5A during the 6-h preincubation led to inhibition of internalization of E. faecalis by both RAW264.7 and peritoneal macrophages (PM). The increase in internalization by Wnt5A signaling (Wnt5A-DMSO versus PBS-DMSO) was nullified by Dsh (Dishevelled) inhibitor, indicating that Dsh is a Wnt5A signaling intermediate (*n* = 6 in RAW264.7, *n* = 4 for PM). PBS and DMSO are vehicle controls for Wnt5A and the inhibitor, respectively. (D) Application of Dsh inhibitor had a similar effect on L. rhamnosus internalization by RAW264.7 and PM (*n* = 4). The CFU count is represented as CFU (× dilution factor). (E to H) Enumeration of the number of cells post-Giemsa staining shows that application of Wnt5A signaling inhibitors (E and F) or actin polymerization inhibitors (G and H) did not lead to notable alteration in the viability of cells of Peyer’s patches (*n* = 15 frames). Data are represented as the mean ± SEM. A *P* value of ≤0.05 was considered statistically significant. *, *P* ≤ 0.05; **, *P* ≤ 0.005; ***, *P* ≤ 0.0005; n, number of experiments. Download FIG S5, TIF file, 0.4 MB. Copyright © 2022 Sengupta et al. 2022 Sengupta et al. https://creativecommons.org/licenses/by/4.0/ This content is distributed under the terms of the Creative Commons Attribution 4.0 International license.

**FIG 6:**
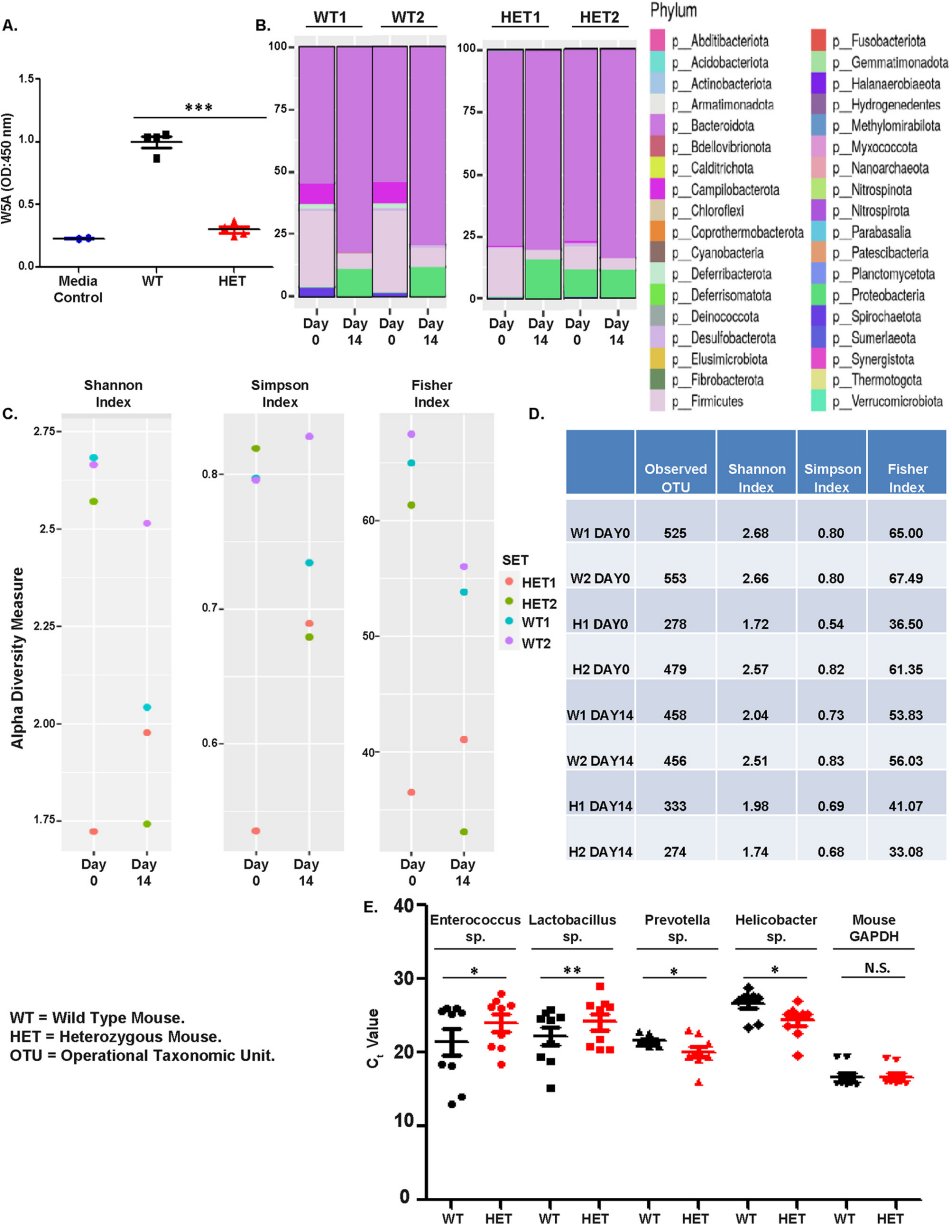
Wnt5A signaling influences colonization diversity of gut commensal bacteria. (A) Lower secretion of Wnt5A from cells of Peyer’s patches of Wnt5A heterozygous mice (lacking a copy of the Wnt5A gene) compared to their wild-type counterparts (*n* = 4 mice). (B) Difference in fecal bacterial phylum abundance between heterozygous mice 1 and 2 with wild-type mice 1 and 2. Observations were made both under normal conditions (day 0) and post-14 days of Abx treatment (day 14). (C) Difference in alpha diversity of the fecal bacterial population of wild-type and heterozygous mice on day 0 and after Abx treatment (day 14) as enumerated using Shannon, Simpson, and Fisher indices. (D) Difference in OTU number enumerated from feces of wild-type (W) and Het (H) mice at day 0 and day14 correlating with the difference in alpha diversity. (E) qPCR from tissue of Peyer’s patches showing the difference in colonization of *Enterococcus*, *Lactobacillus*, *Prevotella*, and *Helicobacter* in the Wnt5A heterozygous and wild-type mice (*n* = 9 mice). A higher *C_T_* value indicates a lower abundance of bacteria in the particular sample. Data are represented as the mean ± SEM. A *P* value of ≤0.05 was considered statistically significant. *, *P* ≤ 0.05; **, *P* ≤ 0.005; ***, *P* ≤ 0.0005.

The influence of Wnt5A on gut bacterial colonization was furthermore reflected in the altered gut bacterial diversity of Wnt5A heterozygous mice, which harbor a single functional copy of the Wnt5A gene and express less than the wild-type level of Wnt5A within the Peyer’s patches (Fig. 6A and Fig. S6) and other tissues (19, 41). Since the bacterial composition of fecal matter is used as a signature of gut bacterial colonization (42–44), we characterized the bacterial population of the feces of Wnt5A heterozygous and wild-type mice as a measure of bacterial colonization therein. Feces were collected both under normal conditions (day 0) and after antibiotic treatment (day 14) from similarly reared wild-type and heterozygous mice (2 from each group) and outsourced for 16S metagenomics sequencing and characterization of bacterial abundance and diversity. Antibiotic was administered specifically to evaluate the potentially protective influence of Wnt5A on gut bacterial abundance/diversity. We found the fecal bacterial colonization of the Wnt5A heterozygous and wild-type mice to be markedly different (Fig. 6B to D). The bacterial presence in the feces of the wild-type and heterozygous mice at both day 0 and day 14 was estimated with respect to a range of different phyla. All phyla present in the different groups (WT: wild type1,2 and HET: heterozygous 1,2) are displayed in the chart and projected as a phylum-abundance plot (Fig. 6B). The related operational taxonomic units (OTU: genus) of the same experimental groups are also projected as alpha diversity using the Shannon, Simpson, and Fisher indices (Fig. 6C and D). These results reveal that gut bacterial colonization is more enriched in the wild-type mice than in the heterozygous mice both under normal conditions (day 0) and after antibiotic treatment (day 14). Additionally, there was a considerable difference in the colonization of the *Enterococcus*, *Lactobacillus*, *Prevotella*, and *Helicobacter* genera within the Peyer’s patches of the Wnt5A heterozygous and wild-type mice as validated by qPCR with specific primers (Fig. 6E). The Peyer’s patches of the Wnt5A heterozygous mice harbored significantly less E. faecalis and L. rhamnosus than those of the wild-type mice, in compliance with the observed dependence of both bacteria on Wnt5A signaling (Fig. 1 and 2). Overall, these results indicate that Wnt5A is a regulator of gut bacterial colonization.

10.1128/msphere.00507-22.6 FIG S6 Wnt5A expression is lower in Peyer’s patches of Wnt5A heterozygous mice compared to those of their wild-type counterparts. (A) Representative confocal microscopy of paraffin section of Peyer’s patch tissue shows lower expression of Wnt5A (green) in heterozygous mice (bottom) compared to wild-type mice (top) (*n* = 2). (B) Wnt5A expression (green) in Peyer’s patches of heterozygous mice was found to be lower than that of their wild-type counterparts even at the cellular level (*n* = 2). n, the number of mice in each group. Download FIG S6, TIF file, 0.4 MB. Copyright © 2022 Sengupta et al. 2022 Sengupta et al. https://creativecommons.org/licenses/by/4.0/ This content is distributed under the terms of the Creative Commons Attribution 4.0 International license.

### Regulation of gut commensal bacterial colonization by Wnt5A signaling is linked with altered major histocompatibility complex (MHC) II expression and T cell activation.

In view of the fact that the gut CD4 T cell repertoire is influenced by the gut-resident commensal bacteria (38, 45, 46), we investigated if the altered gut commensal bacterial colonization that correlates with diminution in Wnt5A signaling affects the gut-associated T cells. To this end, we studied the activation status of T cells in the Peyer’s patches of Wnt5A wild-type and Wnt5A heterozygous mice. Additionally, we analyzed the effect of inhibition of Wnt5A production by IWP-2 on the T cell population and cell surface MHC II expression of isolated Peyer’s patches.

**FIG 7:**
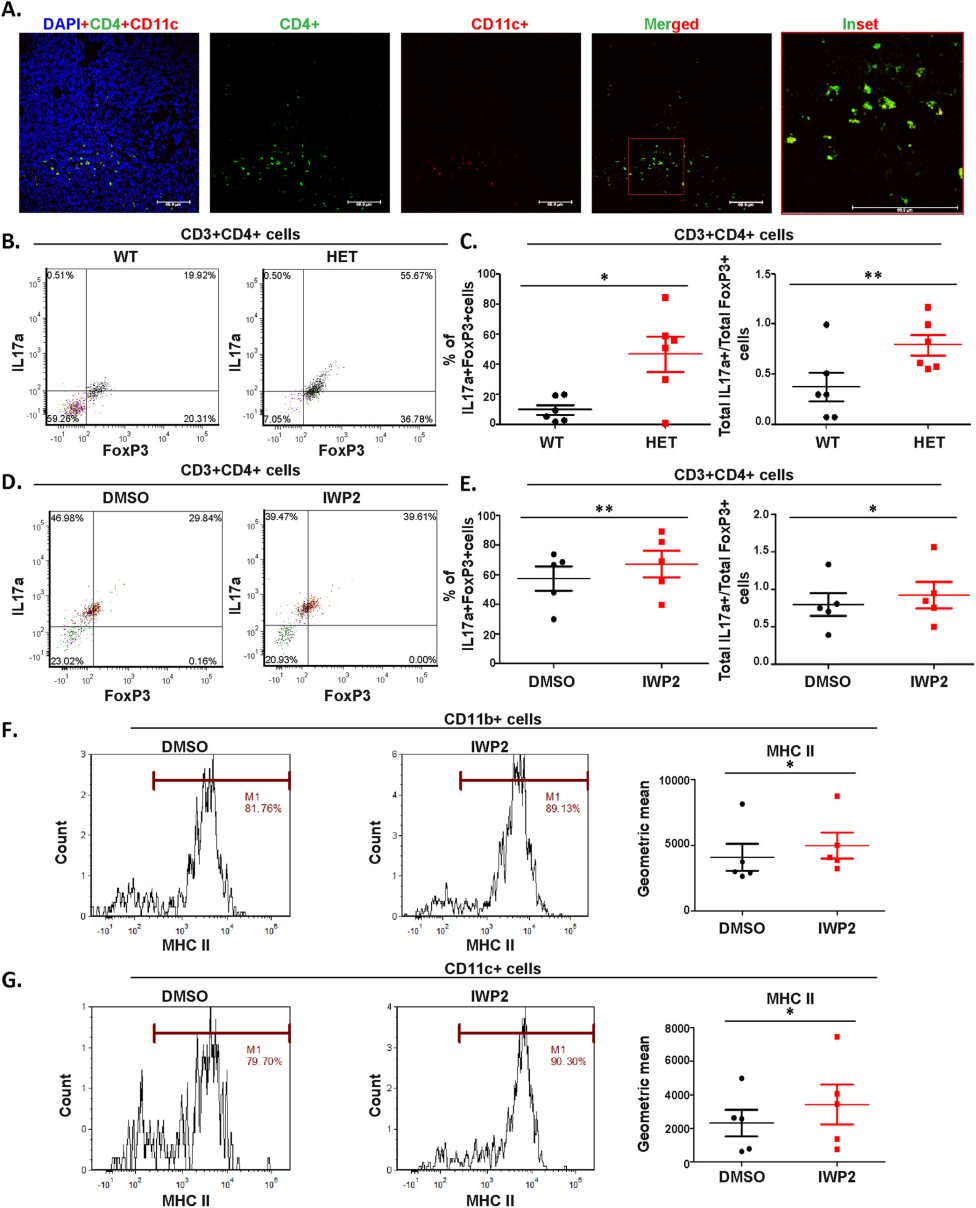
Adaptation of gut commensal bacterial colonization to changes in Wnt5A is linked with altered T cell activation profiles. (A) Colocalization of CD4^+^ T cells (green) and CD11c+ phagocytes (red) in Peyer’s patch tissue visualized by confocal microscopy. The inset shows colocalization (yellow) of both cell types (*n* = 4 mice). (B) FACS representing the difference between the IL17a and FoxP3 expressing CD4^+^ T cell populations in Peyer’s patches of wild-type and heterozygous mice. (C) Plot showing a higher IL17a/FoxP3 ratio, as well as a higher number of IL17a+FoxP3+ CD4 T cells in Peyer’s patches of heterozygous mice lacking a functional copy of the Wnt5A gene as opposed to the wild type (*n* = 6 mice). (D) FACS representing the difference in IL17a+- and FoxP3+-expressing CD4 T cell populations in total cells of Peyer’s patches where Wnt5A signaling was downregulated by IWP2 treatment for 6 h. (E) Plot showing that IWP2 treatment increased the IL17a/FoxP3 ratio and number of IL17a+FoxP3+ CD4 T cells in the total cell population of Peyer’s patches (*n* = 5). (F and G) Higher MHC II surface expression in both CD11b+ (F) and CD11c+ (G) phagocytes of Peyer’s patches upon inhibition of Wnt5A signaling by IWP2 (*n* = 5). Marker M1 indicates the percentage of CD11b+ or CD11c+ cells that were positive for MHC II expression. The markers, quadrants, and gates used for identification were based on the unstained population. Data are represented as the mean ± SEM. A *P* value of ≤0.05 was considered statistically significant. *, *P* ≤ 0.05; **, *P* ≤ 0.005; ***, *P*≤ 0.0005.

Initially, we demonstrated the presence of CD4 T cells in close association with CD11c-expressing phagocytes within the mouse gut Peyer’s patches (Fig. 7A). The CD4 T cell-phagocyte association was similar in both the Wnt5A heterozygous and wild-type mice. However, in concurrence with an overall altered gut commensal bacterial abundance and diversity in comparison to that of the wild-type controls (Fig. 6), the Wnt5A heterozygous mice harbored CD4 T cells with a distinctly different activation profile within the Peyer’s patches of the gut compared to the wild type. As depicted by representative FACS plots (Fig. 7B), FACS analysis of cells of the Peyer’s patches revealed that both the ratio of interleukin 17a+ (IL17a+) to Foxp3+ (IL17a/Foxp3) CD4 T cells and the absolute numbers of IL17a+ Foxp3+ double-positive CD4 T cells are higher in the Peyer’s patches of Wnt5A heterozygous mice than those of the wild type (Fig. 7C). This finding indicated alterations in Foxp3-specific regulatory T cell phenotype, IL17a-specific activated T cell phenotype, and activated versus regulatory T cell differentiation (37, 38, 47–49) within the Peyer’s patches of the Wnt5A heterozygous mice in relation to the wild-type mice. A similar trend toward a relative gain of IL17a-specific activated T cell phenotype with reference to a regulatory FoxP3 phenotype and an increase in IL17a+FoxP3+ T cell numbers was also evident when cells isolated from Peyer’s patches were treated with the Wnt5A production inhibitor IWP-2 (Fig. 7D and E). These results indicate that as Wnt5A signaling shapes the colonization of gut bacterial flora (Fig. 5 and 6), it is also conducive to shaping a bacterial flora-dependent gut CD4 T cell repertoire that ranges from a regulatory to an inflammatory phenotype depending on the Wnt5A signal dosage. Such a concept is in compliance with the presence of CD4 T cells in close association with the phagocytic cells of the Peyer’s patches (Fig. 7A) and the reported existence of a gut flora-specific CD4 T cell repertoire (48–50). The altered MHC II surface expression in the CD11b/CD11c population of the IWP2-treated cells of the Peyer’s patches (Fig. 7F and G) along with IWP2 induced reduction in bacterial numbers (Fig. 5E and F) raised the possibility of bacterial antigen-driven change in MHC II surface expression and CD4 T cell activation. The higher trend of surface MHC II also noted in the cells of the Peyers’s patches of Wnt5A heterozygous mice compared to those of the wild type (Fig. S7) corroborates the possibility of Wnt5A dosage-controlled MHC II-antigen presentation and T cell activation. In compliance with this concept, we have demonstrated differences in the cell surface expression of MHC II molecules as well as CD4 T cell activation in the context of different commensal bacteria (Fig. S8). Other investigators have also discussed MHC II-restricted presentation of bacterial antigens (37). However, the underlying molecular mechanism of such antigen presentation remains elusive. Fig. S9A depicts the gating strategy for IL17a- and Foxp3-positive cells (Fig. 7B to E and Fig. S8C and D), and Fig. S9B depicts the gating strategy for MHC II (Fig. 7F and G, Fig. S7, and Fig. S8A and B).

10.1128/msphere.00507-22.7 FIG S7 Wnt5A heterozygous mice have altered surface expression of MHC II in CD11b/CD11c cells of Peyer’s patches. Heterozygous mice lacking a copy of the Wnt5A gene in correlation to having different commensal bacterial colonization show higher MHC II surface expression in CD11b+ and CD11c+ phagocytes compared to their wild-type counterparts (*n* = 3). Data are represented as the mean ± SEM. A *P* value of ≤0.05 was considered statistically significant. *, *P* ≤ 0.05; **, *P* ≤ 0.005; ***, *P* ≤ 0.0005; n, number of mice in each group. Download FIG S7, TIF file, 0.03 MB. Copyright © 2022 Sengupta et al. 2022 Sengupta et al. https://creativecommons.org/licenses/by/4.0/ This content is distributed under the terms of the Creative Commons Attribution 4.0 International license.

10.1128/msphere.00507-22.8 FIG S8 Commensal bacteria influence MHC II expression in phagocytes and concomitant CD4 T cell activation. (A and B) Enumeration (A) and representative plots (B) showing that infection of RAW264.7 cells by fixed E. faecalis and L. rhamnosus leads to high MHC II surface expression as noted by both the percentage of positive cells and the geometric mean (GM) 12 h postinfection (*n* = 3). (C and D) Graphical illustration (C) and representative quadrant plots (D) demonstrate that CD3^+^ and CD4^+^ T cells from spleen in coculture for 12 h with fixed E. faecalis and L. rhamnosus-infected RAW264.7 cells (3 h postinfection) show a shift towards IL17a+ status compared to regulatory FoxP3+ status. Such exposure also led to a higher number of IL17a+FoxP3+ cells. “Uninfected” indicates phagocytes which went through all similar treatments except exposure to bacteria. Data are represented as the mean ± SEM. A *P* value of ≤0.05 was considered statistically significant. *, *P* ≤ 0.05; **, *P* ≤ 0.005; ***, *P* ≤ 0.0005. The marker gate was based on the unstained population. Download FIG S8, TIF file, 0.1 MB. Copyright © 2022 Sengupta et al. 2022 Sengupta et al. https://creativecommons.org/licenses/by/4.0/ This content is distributed under the terms of the Creative Commons Attribution 4.0 International license.

10.1128/msphere.00507-22.9 FIG S9 Representation of gating strategy for FACS. (A) Gating strategy for detection of IL17a+ and FoxP3+ T cell populations. (B) Gating strategy for observation of MHC II surface expression in CD11c+ and CD11b+ cells. Markers, quadrants, and gates used for identification were based on the unstained population. Download FIG S9, TIF file, 0.2 MB. Copyright © 2022 Sengupta et al. 2022 Sengupta et al. https://creativecommons.org/licenses/by/4.0/ This content is distributed under the terms of the Creative Commons Attribution 4.0 International license.

## DISCUSSION

The requirement of gut commensal bacteria for the maintenance of gut immune homeostasis has been reported extensively (20–22, 51, 52), but the underlying mechanism of bacterial commensalism and immune homeostasis is not clearly deciphered. In this study we explained the importance of steady-state Wnt5A signaling in the safeguarding of certain commensal bacteria within phagocytes. Furthermore, we described that Wnt5A-mediated regulation of commensal bacterial colonization in the murine gut is involved in shaping the gut CD4 T cell repertoire.

Here, we demonstrated that a Wnt5A-actin axis promotes the survival of some common commensal bacteria such as E. faecalis and L. rhamnosus using the macrophage line RAW264.7 and similar phagocytes. Administration of Wnt5A signaling and actin assembly inhibitors to bacteria-harboring phagocytes significantly diminishes the bacterial CFU therein. Both Wnt5A signaling and the phagocyte-resident bacteria enrich a self-sustaining circuit that favors Wnt5A production, maintenance of actin assembly, and bacterial survival (Fig. 1 to 4, Fig. S2). We also demonstrated the presence of intracellular bacteria within the phagocytes of the Peyer’s patches. That Wnt5A signaling supports the survival of certain commensal bacteria, including E. faecalis and L. rhamnosus, within the Peyer’s patches is evident from the significant reduction in the countable bacterial CFU following treatment of the cells of the Peyer’s patches with either IWP-2, which inhibits Wnt5A production (53, 54), or a Dsh-specific inhibitor, which inhibits Wnt5A signaling (31). A similar reduction in the countable bacterial CFU with the application of the actin assembly (Arp2/3) inhibitors suggests that Wnt5A-mediated actin modulation contributes to bacterial colonization (Fig. 5). The regulatory role of Wnt5A in gut bacterial colonization is further corroborated by the notable differences in both fecal and Peyer’s patch-associated bacterial diversity/abundance between the Wnt5A heterozygous mice that lack a functional copy of the Wnt5A gene and their wild-type counterparts. The increased prevalence of *Enterococcus* and *Lactobacillus* in the Peyer’s patches of the Wnt5A wild-type mice compared to the heterozygous mice and the opposite scenario in case of *Prevotella* and *Helicobacter* are quite likely due to variation in the interactions of the existing bacteria with host Wnt5A signaling on account of difference in their pathogenic potential (Fig. 6). In harmony with being conducive to colonization of certain bacterial species in the gut, Wnt5A signaling also supports the maintenance of a gut-specific CD4 T cell repertoire. This is evident from the observed change in cell surface MHC II expression and CD4 T cell activation that correlate with Wnt5A-mediated regulation of gut bacterial colonization (Fig. 7, Fig. S7).

In light of the existing literature describing commensal bacteria-specific CD4 T cells (22, 37), it is quite possible that the Wnt5A-actin axis of the phagocytes of the Peyer’s patches orchestrates presentation of resident bacteria-derived antigens to CD4 T cells, thereby regulating their activation. This concept is in compliance with the presence of bacteria-harboring phagocytes in coexistence with CD4 T cells within the Peyer’s patches (Fig. 5G and 7A) and the demonstrated activation of CD4 T cells in coculture with bacteria-harboring phagocytes (Fig. S8). Depending on the extent and affinity of MHC II-bacterial peptide-CD4 T cell receptor interactions, the CD4 T cells could be accordingly sensitized at the transcriptional level, leading to the development of an activated (for example, IL17a secreting) or regulatory (for example, Foxp3 expressing) phenotype (22, 55). Thus, the Wnt5A-actin axis of phagocytes could play a major role in shaping the gut steady-state CD4 T cell repertoire in the context of the incumbent bacteria.

This study upholds the requirement of Wnt5A signaling as a crucial regulatory factor of host-commensal mutualism and gut immune homeostasis, lack of which may result in dysbiosis and the development of autoimmune disorders (24, 56). However, how Wnt5A signaling controls the distribution of intracellular, mucosal, and lumen-associated gut bacterial populations remains unclear. Investigation into the involvement of Toll-like receptor (TLR), nucleotide-binding oligomerization domain 2 (NOD2) and other pattern recognition receptors (56–61) in the complex network of commensalism and T cell homeostasis, which should be of considerable interest, has also not been addressed here.

## MATERIALS AND METHODS

### Cells.

The RAW264.7 cell line was obtained from ATCC (ATCC TIB71). Mouse peritoneal macrophages and cells of Peyer’s patches were harvested and cultured following previously published protocols (17, 19, 62–64).

### Reagents.

The list of reagents used is provided in Table 1.

**TABLE 1:** List of reagents used in the study^*a*^

Reagent or Resource	Source	Ref. no.
DMEM high glucose	Life Technologies (Thermo Fisher Scientific, USA)	12800-017
RPMI 1640	Life Technologies (Thermo Fisher Scientific, USA)	31800-022
HI FBS	Life Technologies (Thermo Fisher Scientific, USA)	10082147
Penicillin-streptomycin	Life Technologies (Thermo Fisher Scientific, USA)	15140122
l-glutamine	Life Technologies (Thermo Fisher Scientific, USA)	25030081
1× trypsin EDTA	Life Technologies (Thermo Fisher Scientific, USA)	25200-072
Trizol reagent	Life Technologies (Thermo Fisher Scientific, USA)	15596018
DAPI	Life Technologies (Thermo Fisher Scientific, USA)	D1306
Alexa Fluor 555 phalloidin	Life Technologies (Thermo Fisher Scientific, USA)	A34055
Brain heart infusion broth (BHI)	HiMedia Laboratories, India	M210
MRS broth	HiMedia Laboratories, India	M369
Agar agar	HiMedia Laboratories, India	GRM666
Anti-β actin antibody	Santa Cruz Biotechnology, USA	SC-47778
IWP2	Santa Cruz Biotechnology, USA	SC-252928
Anti-mouse Wnt5A antibody	R&D Systems, USA	MAB-645
Anti-mouse Wnt3A antibody	R&D Systems, USA	MAB-1324
Anti-rat HRP conjugated 2° antibody	R&D Systems, USA	HAF005
Anti-mouse CD4	R&D Systems, USA	MAB-554
Anti-mouse HRP conjugated 2° antibody	Sigma-Aldrich, USA	A9044
Collagenase D	Sigma-Aldrich, USA	11088866001
Kanamycin sulphate	Sigma-Aldrich, USA	K4000
Gentamycin	Sigma-Aldrich, USA	G1914
Colistin	Sigma-Aldrich, USA	C4461
Metronidazole	Sigma-Aldrich, USA	M1547
Vancomycin	Sigma-Aldrich, USA	V2002
Lysozyme	Sigma-Aldrich, USA	L6876
Glycerol	Sigma-Aldrich, USA	G5516
NaCl	Sigma-Aldrich, USA	S5886
NP-40	Sigma-Aldrich, USA	492018
Triton X-100	Sigma-Aldrich, USA	11332481001
Anti-Fz5 antiserum, raised in rabbit	BioBharati Life Sciences, India	
CDNA synthesis kit	BioBharati Life Sciences, India	BB-E0043
Taq polymerase	BioBharati Life Sciences, India	BB-E0010
DMSO	MP Biomedicals, USA	196055
rWnt5A	Merck, Germany	GF146
Rac1 inhibitor	Merck, Germany	NSC23766
Disheveled (Dsh-PDZ domain) inhibitor	Merck, Germany	CAS294891-81-9
Arp 2/3 complex inhibitor I	Merck, Germany	CK-666
Arp 2/3 complex inhibitor II	Merck, Germany	CK-869
Tris base	Merck, Germany	648310
Na_3_VO_4_	Merck, Germany	D00152519
TMB solution	Merck, Germany	CL07-1000MLCN
PVDF membrane	Millipore, USA	IPVH00010
Lumintaclassico chemiluminescent substrate	Millipore, USA	WBLUC0500
MgCl_2_	Millipore, USA	60583305001046
Tween-20	Millipore, USA	655205
β-mercaptoethanol	Millipore, USA	8057400250
NaF	Millipore, USA	61773705001730
SMART pool mouse Wnt5A siRNA	Dharmacon, USA	L-065884-01
SMART pool mouse Wnt3A siRNA	Dharmacon, USA	046386-00-0005
Nontargeting pooled control siRNA	Dharmacon, USA	D-001810-05
V500 Syrian hamster anti-mouse CD3	BD Biosciences, USA	560771
PerCP-Cy 5.5 rat anti-mouse CD4	BD Biosciences, USA	550954
BV605 rat anti-mouse CD8	BD Biosciences, USA	563152
Alexa Fluor 488 rat anti-mouse IL17a	BD Biosciences, USA	560221
Alexa fluor 647 rat anti-mouse FoxP3	BD Biosciences, USA	560401
FITC rat anti-mouse CD11b	BD Biosciences, USA	553310
PE rat anti-mouse CD11b	BD Biosciences, USA	557397
APC hamster anti-mouse CD11c	BD Biosciences, USA	550281
PE mouse anti-mouse H-2K[d]	BD Biosciences, USA	553566
PerCP-Cy 5.5 rat anti-mouse I-A/I-E	BD Biosciences, USA	562383
FITC mouse anti-mouse H-2Kb	BD Biosciences, USA	553569
Propidium iodide	BD Biosciences, USA	556463
Fixable viability stain 450	BD Biosciences, USA	562247
MS column	Miltenyi Biotec, Germany	130-042-201
CD4^+^ T Cell isolation kit, mouse	Miltenyi Biotec, Germany	130-104-454

a HRP, horseradish peroxidase; PVDF, polyvinylidene difluoride; FITC, fluorescein isothiocyanate; PE, phycoerythrin; APC, allophycocyanin.

### Animal maintenance.

The institute animal facility was used for breeding and maintenance of BALB/c mice and B6;129S7-*Wnt5a^tm1Amc^*/J mice (Wnt5A+/+ and Wnt5A+/–), purchased from Jackson Laboratory, USA. Wnt5A+/+ and Wnt5A+/– mice, which were littermates, were characterized by genotyping, using the Jackson laboratory protocol (https://www.jax.org/Protocol?stockNumber=004758&protocolID=23556). Mice were maintained in individually ventilate cages (IVCs) under optimum conditions with controlled food/water supply and light/dark cycles. Experimental and control mice, 8 to 10 weeks old, were used in similar male:female ratios.

### Isolation and characterization of commensal bacteria.

Commensal bacteria were isolated from the cecum of a BALB/c mouse by perforation with a needle. BHI broth was inoculated with the needle and incubated overnight before plating in BHI agar. After 12 h colonies were plucked, from which pure cultures were isolated for 16S sequencing. E. faecalis was characterized thereafter. L. rhamnosus and S. enterica were purchased from MTCC, Chandigarh, India (MTCC 1408^T^, MTCC 3224).

### Isolation and culture of cells from Peyer’s patches.

The small intestine was harvested from BALB/c or Wnt5A wild-type (+/+) and heterozygous (+/–) mice as needed and flushed with sterile PBS to remove fecal material. Peyer’s patches were identified opposite to the mesenchymal end of the intestine, harvested as sections of 1-mm length, and shaken at 200 rpm at 37°C in RPMI 1640 medium supplemented with 10% fetal bovine serum (FBS) and 10 mM EDTA for 20 min. After decanting the medium and replenishing it with fresh medium containing collagenase D and DNase I (AM2222), the material was shaken for an additional 15 min. Following passage of dispersed tissue through a 40-μM cell strainer, the mixture was again shaken at 200 rpm for 10 min and subsequently passed through another 40-μm strainer to get a single cell population for use in flow cytometry or culturing as needed.

### Bacterial uptake and survival assays.

For uptake assays, RAW264.7 or mouse peritoneal macrophages pretreated with rWnt5A (50 ng/mL, dissolved in PBS) for 6 h were infected with either E. faecalis or L. rhamnosus at a multiplicity of infection (MOI) of 10 and 20, respectively, for 2 h in high glucose dulbecco’s modified eagle's medium (DMEM) without antibiotic and FBS. Cells were then washed with PBS, lysed in sterile water, and plated in either BHI (E. faecalis) or MRS (L. rhamnosus) agar. CFU were counted after 14 h (BHI) or 36 h (MRS) of incubation at 37°C with (MRS) or without (BHI) 5% CO_2_. Data presented as log CFU were enumerated as log (CFU × dilution factor).

For survival assays, similarly treated cells were infected with E. faecalis (MOI, 10) or L. rhamnosus (MOI, 20) for 1 h. External bacteria were washed with PBS, and the cells were either harvested to estimate the CFU (time point *T*0) or incubated with DMEM high glucose without antibiotic, to be harvested at 1 h intervals (*T*1 to *T*4) for assessing survival of the internalized bacteria at the different time points. Lysis, plating, and CFU counting were done as before.

For observing the effect of Dsh (15 μm), Rac-1 (15 μm), or actin polymerization inhibitors CK 666 and CK 869 (20 μm each) on bacterial survival, infected cells were incubated in antibiotic-free DMEM in the presence of the desired inhibitors for 3 h (*T*3) followed by harvesting, plating, and CFU enumeration. In the case of Fz5 antiserum application, the same experiments were performed, with the only exception being that cells were harvested 3 and 6 h (*T*3, *T*6) postinfection.

For bacterial uptake/survival assays with cells obtained from Peyer’s patches, incubation for 12 h in RPMI 1640 plus antibiotic medium was done to minimize cell-associated bacteria.

Survival of endogenous bacteria of Peyer’s patches was estimated after isolation and culture of cells for 6 h at 37°C without antibiotics separately with Dsh inhibitor (15 μM), IWP2 (0.05 μM), CK666 (20 μM), CK869 (20 μM), or DMSO-vehicle control.

### Transfection.

RAW264.7 macrophages were plated for 6 to 8 h in six-well tissue culture plates (approximately 2 × 10^6^ cells per well) and incubated at 37°C with 5% CO_2_ and subsequently infected with either E. faecalis or L. rhamnosus at an MOI of 10 or 20 for 1 h. After removal of the external bacteria, the infected cells were incubated for 2 h in DMEM complete medium to ensure removal of extracellular bacteria. Subsequently, the medium was replaced by transfection mix as follows. Wnt5A siRNA (50 μM) or Wnt3A siRNA (100 μM) or nontargeting (control) siRNA(100 μM) separately mixed with 5 μL lipofectamine RNAiMAx reagent in 300 μL of antibiotic-free serum-free OPTI-MEM medium was incubated for 30 min, diluted to 25 nM in 700 μL antibiotic-free DMEM with 2% FBS, and added to the cells in each well. After 24 h the culture was replaced with DMEM complete medium and incubated further for 36 h, following which washing, lysis, and plating were done. CFU were calculated controlled to cell number.

### Western blotting.

Western blotting was done following published protocols (19). Visualization of the membrane was done with chemiluminescent substrate using Chemi documentation systems from Invitrogen (iBright FL-1500) and Azure Biosystems (model-C400). Band intensities were calculated utilizing GelQuant.Net.

### Confocal/fluorescence microscopy.

**(i) Propidium iodide (PI) staining.** For identification of intracellular bacteria, propidium iodide staining was performed following a previously published protocol (19). Observation was done under a fluorescence microscope (Leica DMI3000 B) at 100× objective and ×1 zoom.

**(ii) Phalloidin staining.** Phalloidin binding to assembled actin of RAW264.7 cells was visualized after following a previously published protocol (19) under a Zeiss LSM 980 microscope at ×60 magnification and ×2 zoom. Transfected cells were treated similarly for microscopy and visualized using either the Zeiss LSM 980 or Leica TCS-SP8 microscope at ×60 magnification and ×2 zoom.

**(iii) Antibody staining of paraffin section of Peyer’s patches.** Peyer’s patches were isolated and fixed overnight in 10% buffered formalin and embedded in paraffin for making 3-μm sections by microtome. Sections were then deparaffinized and rehydrated using the following treatment of slides: xylene 2 × 3 min, xylene 1:1 with 100% ethanol for 3 min, 100% ethanol 2 × 3 min, 95% ethanol for 3 min, 70% ethanol for 3 min, 50% ethanol for 3 min, and lastly, rinse with cold tap water. Heat-induced epitope retrieval was done using Tris-EDTA buffer (10 mM Tris base, 1 mM EDTA, 0.05% Tween 20, pH 9.0). Antibody staining was done using 1% BSA in Tris-buffered saline (TBS). Primary antibody incubation was overnight at 4°C, followed by incubation with Alexa Fluor 488-conjugated secondary antibody for 2 h at room temperature. For fluorophore-conjugated primary antibodies, staining was done for 2 h at room temperature. Tissue sections were visualized under a Leica TCS-SP8 microscope at ×40 magnification and ×0.75 zoom.

### Flow cytometry.

For checking intracellular IL-17 or Foxp3, cells isolated from Peyer’s patches were allowed to settle and were treated with 3 μg/mL brefeldin A for 4 h. After washing with PBS, cells were fixed with 1% paraformaldehyde and permeabilized with PBST (0.5% Tween 20). Subsequently, antibody staining was done in 1% BSA in PBST for 1 h at 4°C. For observation of surface MHC II expression, live cells without brefeldin A exposure were stained. Flow cytometry was done with a BD.LSR Fortessa cell analyzer, and analysis was done using FCS Express 5 software from BD (Beckton Dickinson).

### *In vitro* coculture studies.

RAW264.7 macrophages were either infected with fixed E. faecalis and L. rhamnosus for 1 h at an MOI of 10 or 20, respectively or left untreated. Then, 3 h postinfection, CD4^+^ T cells harvested from the spleen of BALB/c mice and purified by magnetic separation were added to either the infected or uninfected culture. Next, 12 h into the coculture, 3 μg/mL brefeldin A was added, and after 4 h, cells were harvested and processed to check IL17a and FoxP3 by flow cytometry.

### Analysis of fecal bacterial diversity.

Fecal pellets were collected from mice without any treatment or after 14 days of Abx cocktail (4 mg/mL kanamycin, 0.35 mg/mL gentamicin, 8,500 U/mL colistin, 2.15 mg/mL metronidazole, and 0.45 mg/mL vancomycin) (65) treatment and sent for 16S sequencing and data analysis to LCGC Life Sciences/WIPRO. Quality assessments of the isolated genetic materials were done using D1000 ScreenTape assay of TapeStation systems. The Size range was 35 to 1,000 bp with 15% and 10% resolution between 35 to 300bp and 300 to 1,000 bp. Trimming of adapters and poor read sequences was done using Trim Galore. Quality profiling and error estimation of sequence reads was conducted utilizing the R package dada2. The Kraken 2 and Bracken tools were used to align the filtered reads in relation to 16S rRNA annotations from the Silva v138 database. Phylogenetic analysis was done generating plots and statistics for taxonomic classification with the R package phyloseq. Visualization of the interactive hierarchical chart representing taxonomic classification was done using the Krona tool.

### RNA isolation for qPCR.

Peyer’s patches were excised, cleaned with PBS, and homogenized in 600 μL TRIzol 2 to 3 times for 2 min on ice. Then, 120 μL of chloroform was added to each homogenate and mixed well, followed by centrifugation at 16,000 × *g* for 12 min to collect the upper transparent phase. Next, 300 μL of isopropanol was added and mixed, and after incubation on ice for 30 min, centrifugation was performed at 16,000 × *g* for 12 min. Supernatant was discarded, and the pellet was washed with 70% ethanol and left to air dry. Dried RNA pellet was dissolved in diethyl pyrocarbonate (DEPC)-treated water by heating at 50°C. Gene-specific cDNA was made with a standard reverse transcriptase (RT) kit (BioBharati Life Sciences) using reverse primers specific to 16S genes of the targeted bacterial strains. qPCR was run in CFXOpus96 (Bio-Rad). Amplification was visualized using SYBR green dye. Threshold cycle *C_T_* values were plotted using GraphPad Prism 5 for statistical analysis. The list of primers used is provided in Table 2.

**TABLE 2:** List of qPCR primers used in the study

Organism	Primer name	Primer sequence (5′–3′)	No. of bases
Enterococcus spp.	EF_ID_Fw	GCAAGTCGAACGCTTCTTTC	20
	EF_ID_Rv	GCACCTGTTTCCAAGTGTTATC	22
Lactobacillus spp.	Lac_ID_Fw	GAAGGCTTTCGGGTCGTAAA	20
	Lac_ID_Rv	CGTGGCTTTCTGGTTGGATA	20
Prevotella spp.	Prev_ID_Fw	GATGCGTCTGATTAGCTTGTTG	22
	Prev_ID_Rv	CCGTGTCTCAGTTCCAATGT	20
Helicobacter spp.	Hel_ID_Fw	GGAATCACTGGGCGTAAAGA	20
	Hel_ID_Rv	CCTCTCCCACACTCTAGACTAATA	24
Mouse	Mo_RNA_GAPDH_Fw	AACAGCAACTCCCACTCTTC	20
	Mo_RNA_GAPDH_Rv	CCTGTTGCTGTAGCCGTATT	20

### ELISA.

Indirect ELISA to estimate Wnt5A or Wnt3A secreted from RAW264.7 cell culture (infected or uninfected) was done by harvesting and centrifugation of culture media using the appropriate antibodies (19).

### Statistical analysis.

Statistical analysis was done utilizing paired or unpaired Student’s *t* test as needed in GraphPad Prism 5 software. Graphs and line diagrams are represented as the mean ± standard error of the mean (SEM), and a *P* value of ≤0.05 was considered statistically significant. Significance is represented by asterisks (*) in the following manner: *, *P* ≤ 0.05; **, *P* ≤ 0.005; ***, *P* ≤ 0.0005.

### Ethics statement.

All animal experiments were conducted with approval of Animal Ethics Committee of CSIR-IICB as per meeting IICB/AEC/Meeting/Sep/2019/1 held on 19 September 2019.
