# Supplementary figures and images for: Wnt5A Signaling Regulates Gut Bacterial Survival and T Cell Homeostasis
Source: mSphere. 2022 Dec 6;7(6):e00507-22. doi: 10.1128/msphere.00507-22 (PMC9769580; doi:10.1128/msphere.00507-22)

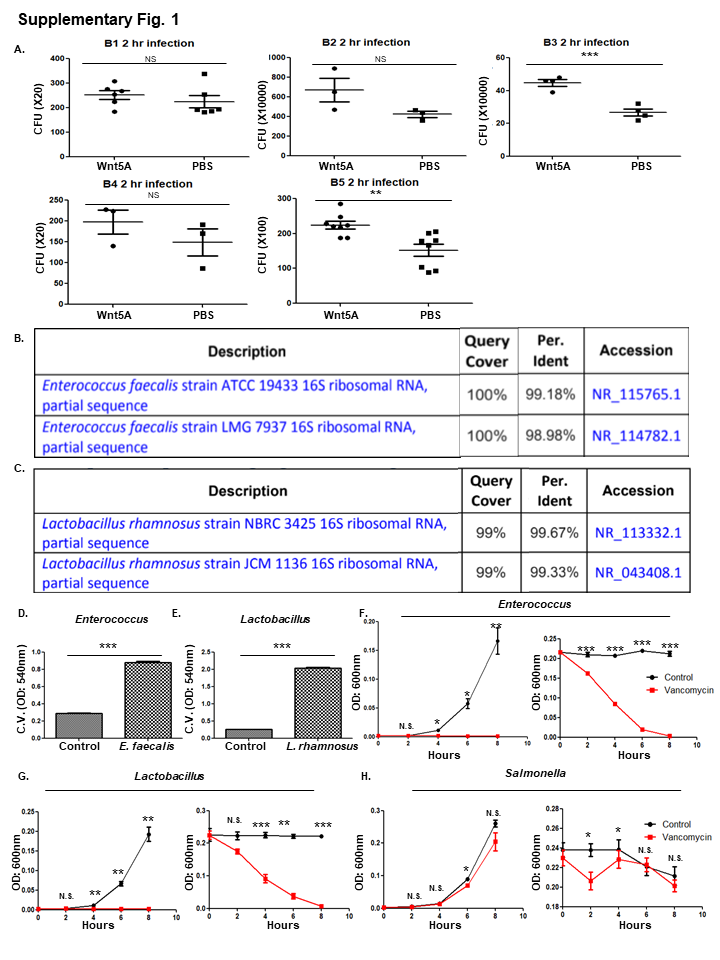

Supplement: FIG S1 [file msphere.00507-22-s0001.tif]

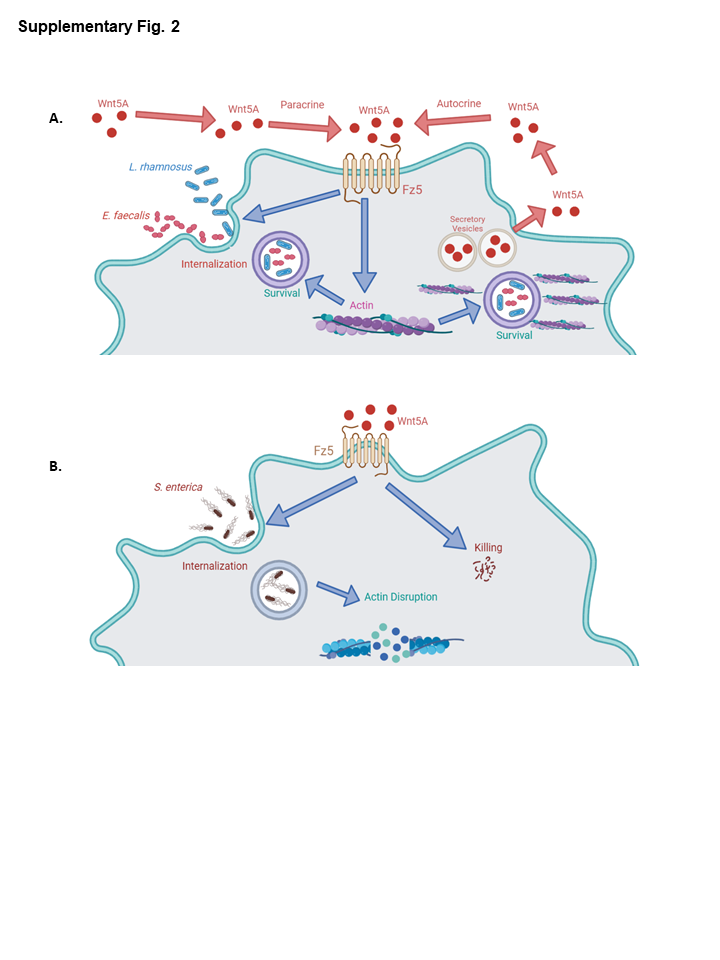

Supplement: FIG S2 [file msphere.00507-22-s0002.tif]

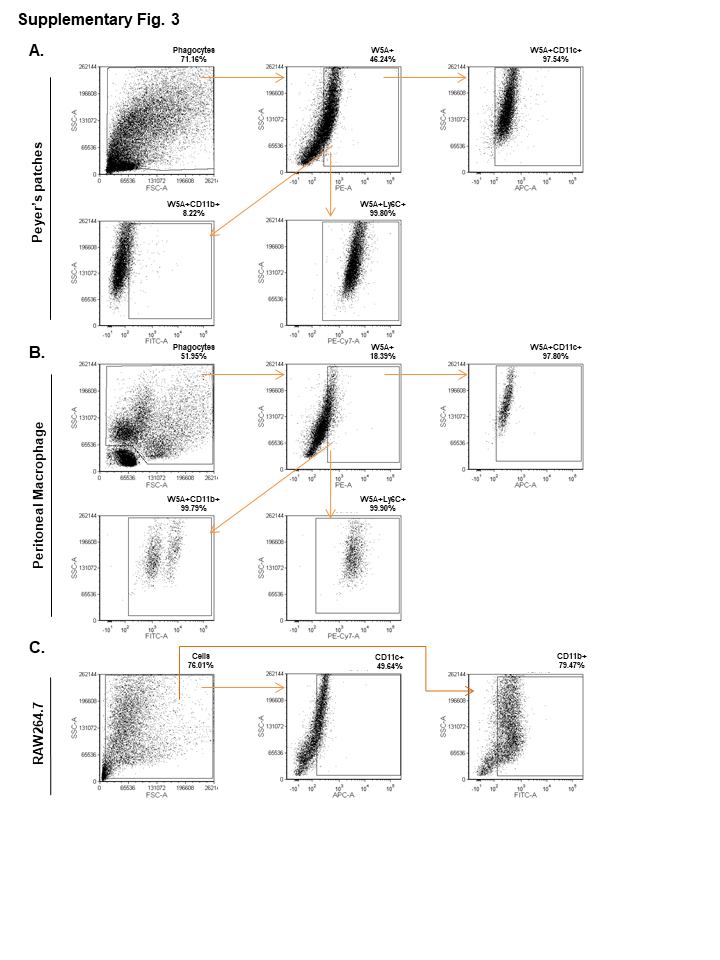

Supplement: FIG S3 [file msphere.00507-22-s0003.tif]

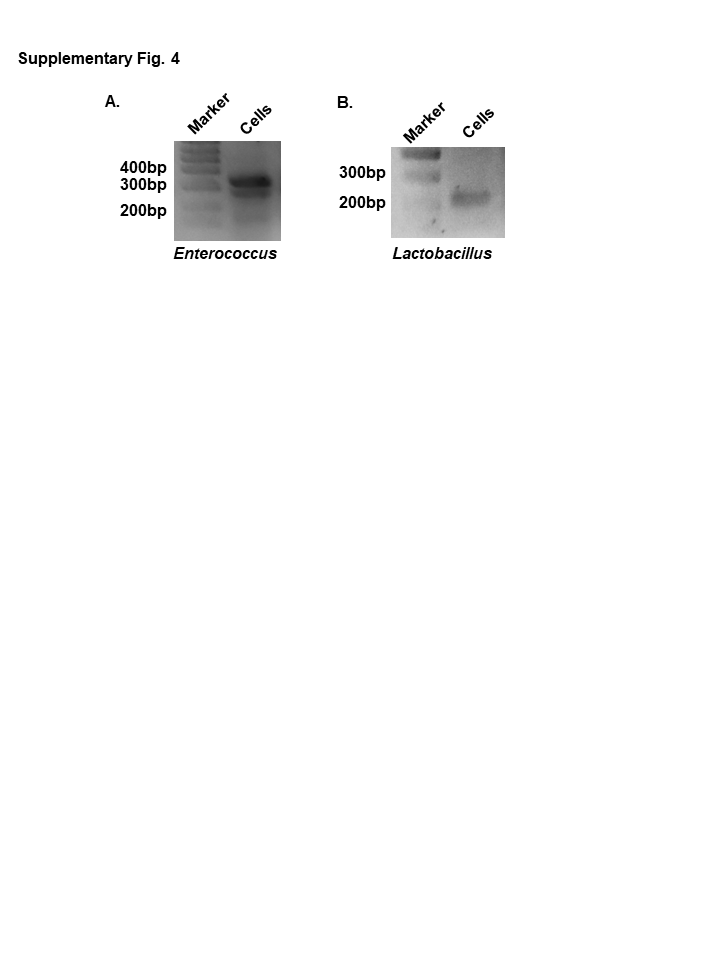

Supplement: FIG S4 [file msphere.00507-22-s0004.tif]

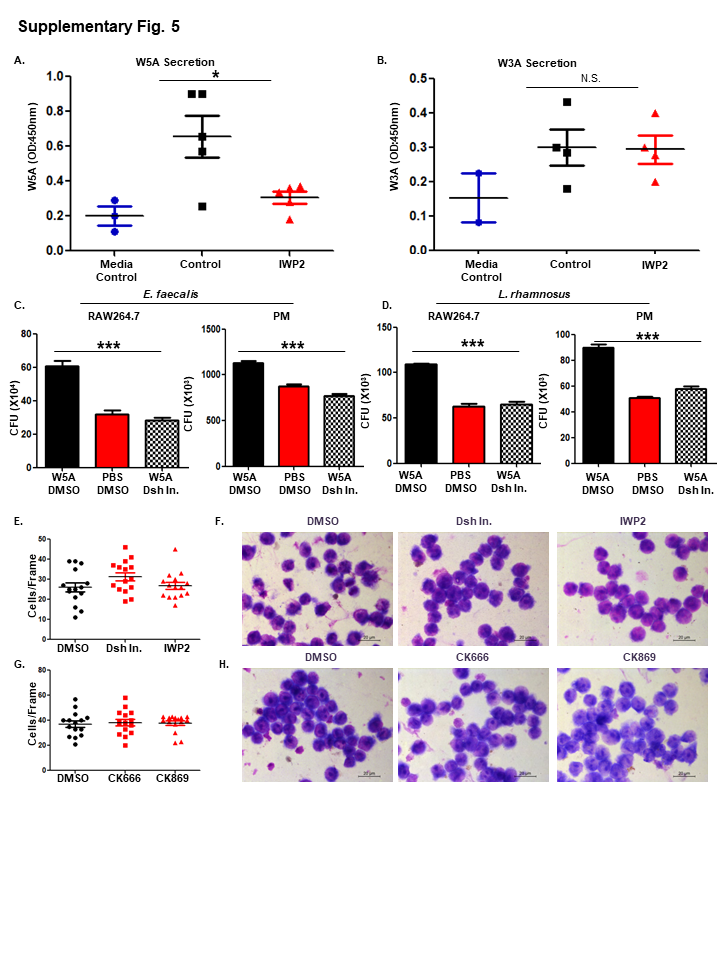

Supplement: FIG S5 [file msphere.00507-22-s0005.tif]

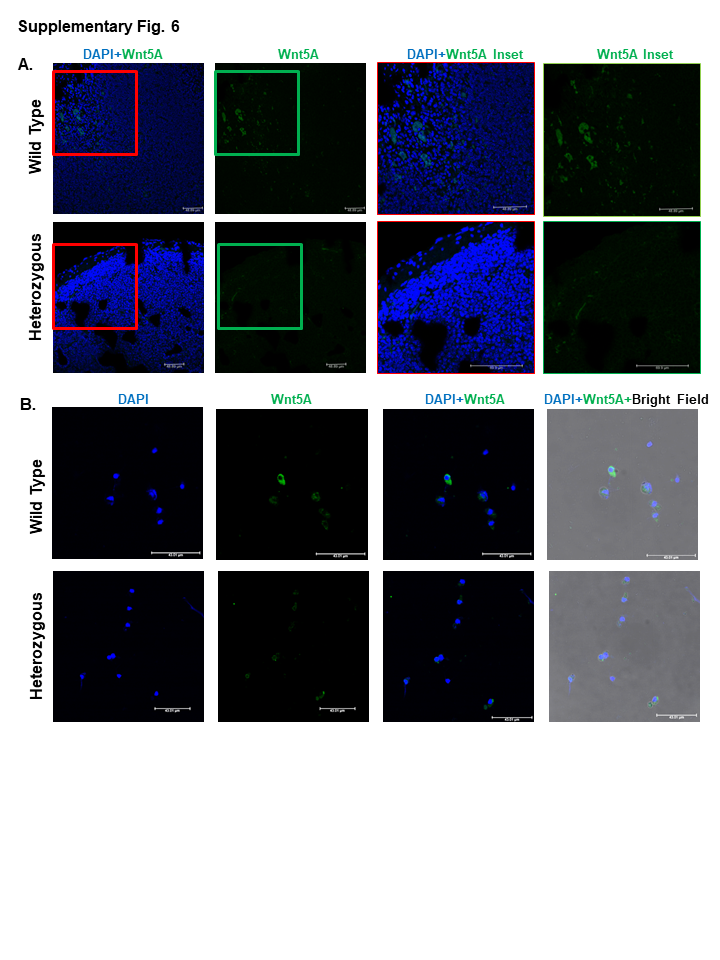

Supplement: FIG S6 [file msphere.00507-22-s0006.tif]

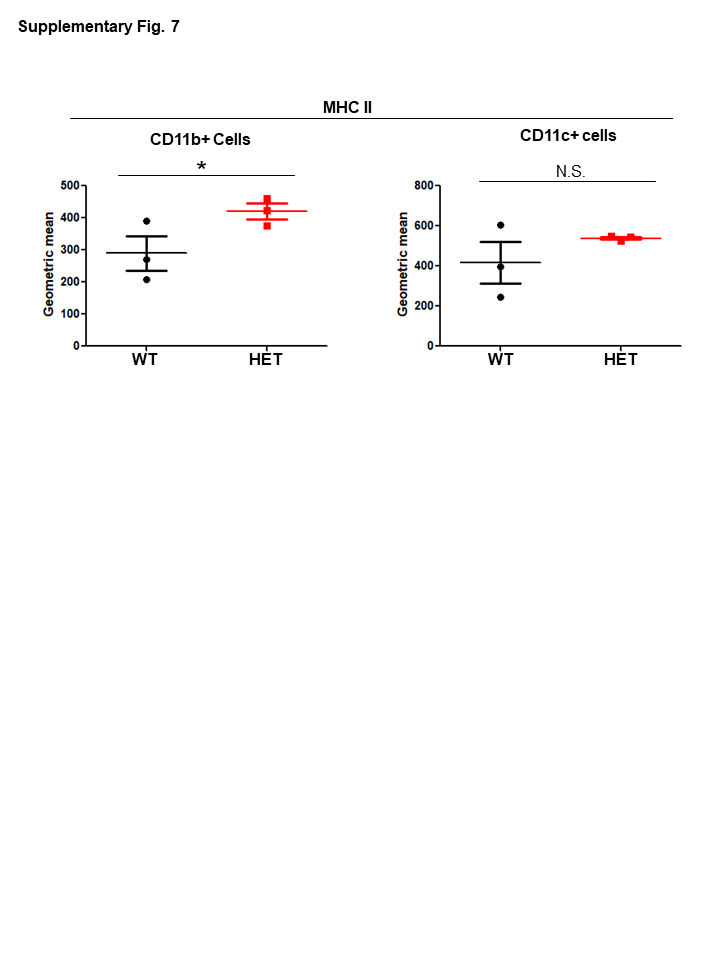

Supplement: FIG S7 [file msphere.00507-22-s0007.tif]

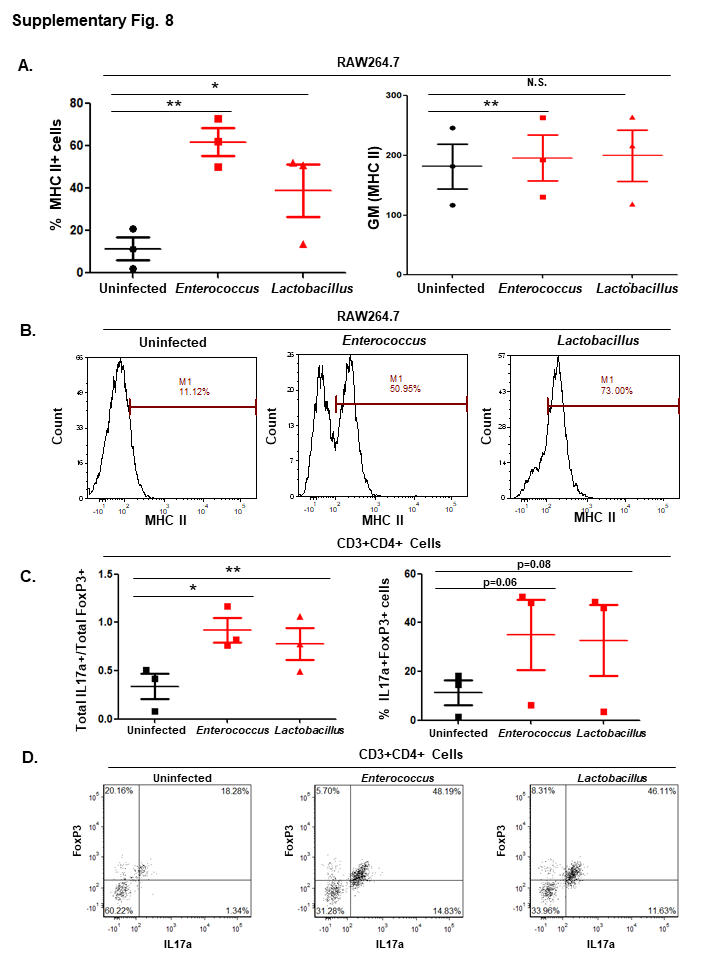

Supplement: FIG S8 [file msphere.00507-22-s0008.tif]

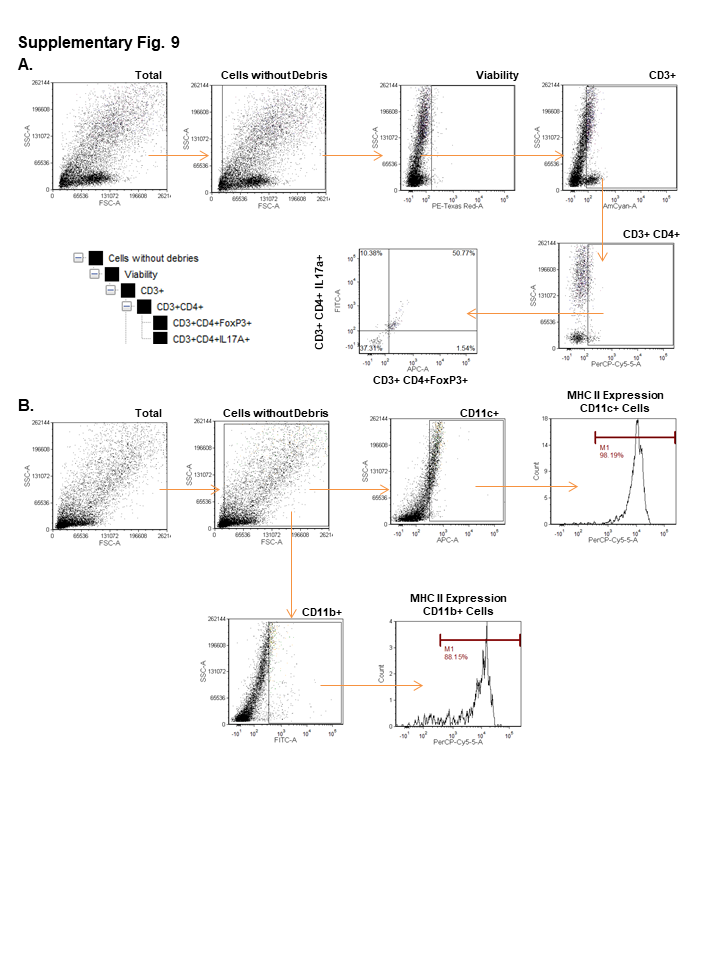

Supplement: FIG S9 [file msphere.00507-22-s0009.tif]
